# Karyotype Analysis, Genomic and Fluorescence In Situ Hybridization (GISH and FISH) Reveal the Ploidy and Parental Origin of Chromosomes in *Paeonia* Itoh Hybrids

**DOI:** 10.3390/ijms231911406

**Published:** 2022-09-27

**Authors:** Litao Cui, Tai Chen, Xin Zhao, Shunli Wang, Xiuxia Ren, Jingqi Xue, Xiuxin Zhang

**Affiliations:** Institute of Vegetables and Flowers, Chinese Academy of Agricultural Sciences, Key Laboratory of Biology and Genetic Improvement of Horticultural Crops, Ministry of Agriculture and Rural Affairs, Beijing 100081, China

**Keywords:** *Paeonia* Itoh hybrids, intersectional hybrids, ploidy, karyotype, GISH, FISH, 5S rDNA

## Abstract

Itoh hybrids are intersectional hybrids in *Paeonia* L. with sect. *Moutan* and sect. *Paeonia* as paternal and maternal parents, respectively. Therefore, these hybrids have herbaceous stems with improved ornamental value introduced by the paternal parent. Although both of their parents are diploids, Itoh hybrids are triploids. Moreover, the parental origin of their chromosomes has not been extensively studied. This study systematically analyzed the genome size, ploidy, and karyotype of Itoh hybrids and compared them with their parental taxa. Although the monoploid genome size of Itoh hybrids was different, it was not significantly different from that of the parents. However, the size of varieties in the two parental taxa was significantly different from the wild species, probably due to genome rearrangements caused by artificial selection. Further karyotype analysis, correlation analysis, and hierarchical clustering could not identify the parental origin of chromosomes in Itoh hybrids. Verification through genomic and fluorescence in situ hybridization (GISH and FISH) suggested that for the three sets of chromosomes in Itoh hybrids, two were from the paternal parent, and one was from the maternal parent. One of the first two sets was from wild species, and the other from a cultivated variety. GISH could not label the chromosomes of cultivated peonies from the sect. *Moutan*, probably due to the huge and complex genomes compared with the wild species. Meanwhile, 5S rDNA-based FISH was first applied in *Paeonia*, which may be used for ploidy assessment. This work may give insights into the utilization of Itoh hybrid resources.

## 1. Introduction

Peony (*Paeonia* L.), both tree and herbaceous, is one of the most important ornamental plants worldwide, with high economic and humanistic values. Tree peony has over 1000 cultivars and is widely used as garden or pot flowers [1]. Herbaceous peony is common in the cut-flower market, especially as a love flower [2]. *Paeonia* L. is the only genus of Paeoniaceae and has around 40 species, which can be further divided into three sections (*Moutan*, *Paeonia*, and *Onaepia*). Sect. *Moutan* has nine species (tree peonies) native to China. Sect. *Paeonia* is the largest section and has about 27 species (herbaceous peonies), mainly native to Asia and Europe. Sect. *Onaepia* has only two species (herbaceous peonies) native to North America [3,4]. In addition to tree and herbaceous peonies, the Itoh hybrid is another hybrid peony group. The Itoh hybrid is the intersectional hybrid of sect. *Moutan* and sect. *Paeonia*, as paternal and maternal parents, respectively, and was first cultivated by Toichi Itoh in 1948. All Itoh hybrids are herbaceous as the stem grows quickly in spring and dies in autumn. They look more similar to tree peony with increased color range and flares at the petal base and are thus preferred for cut flowers [5].

Chromosomes (present as a pair) are the main carriers of genetic information in flowering plants. Polyploidy (whole genome duplication), which refers to the repetition of all sequences in the genome, provides the original genetic material for biological evolution and makes the plant genome recombine rapidly. Chromosomes undergo diploidization in most plants during evolution, thus forming polyploids (autopolyploid and allopolyploid). Autopolyploids are via intraspecific genome duplication, which contain chromosomes from the same species, while allopolyploids are via the merging of distinct species through hybridization and subsequent genome duplication, which contain chromosomes from different species [6]. In addition to diploidy, tetraploidy and hexaploidy are also common in natural or artificially selected varieties. They usually have enlarged organs for improving the agronomic traits in many crops and ornamental value in ornamental plants, such as tetraploid potato (*Solanum tuberosum* L.) and cotton (*Gossypium* spp.) [7,8], hexaploid wheat (*Triticum aestivum* L.) and chrysanthemum (*Chrysanthemum morifolium* Ramat.) [9,10], and octoploid strawberry (*Fragaria* × *ananassa*) and triticale (×*Triticosecale* Wittmack) [11,12]. The odd polyploid varieties are also domesticated for special breeding purposes, such as triploid seedless watermelon [13].

Karyotype analysis is a traditional cytogenetic method that compares chromosome morphology and number among different species or varieties to provide insight into the origin and evolution of plant species, molecular phylogeny, and floristic geography. It also classifies plant species rapidly, as well as confirming alien disomic addition lines in breeding by identifying several basic cytological parameters [14,15,16]. The number of chromosomes and karyotype of a species or variety is usually stable. Differences in these parameters between populations usually lead to reproductive isolation in most cases, maintaining the purity and stability of a species. Arm ratio, the longest chromosome to the shortest chromosome ratio, represents the basic karyotype of a cultivar. The combination of arm ratio and the proportion of chromosomes with chromosome arm ratio less than 2:1 refers to a karyotype classification known as Stebbins’s classification [17]. Chromosome arm ratio, the long arm to the short arm of each chromosome, is also one of the key chromosome traits. Based on this parameter, the chromosome can be divided into four types: (1) metacentrics, the centromere is in the median point (M, 1.0) or median region (m, 1.0–1.7); (2) submetacentrics, the centromere is in the submedian region (sm, 1.7–3.0); (3) subtelocentrics, the centromere is in the subterminal region (st, 3.0–7.0); (4) telocentrics, the centromere is in the terminal region (t, 7.0–∞) or terminal point (T, ∞) [18].

So far, karyotype analysis has been wildly used for species identification, cytotaxonomy study, and molecular phylogenetic analysis in plant and other organisms [15,19,20,21,22,23]. Although the morphology-based karyotype analysis is simple and easy to operate, it has some limitations. For example, it cannot distinguish between individual chromosomes with similar morphology, size, and parental origin from a pair of chromosomes [24]. As a result, genomic in situ hybridization (GISH) on plant chromosomes has been introduced into molecular cytogenetics, in which a specific genomic DNA, i.e., one of the parents of hybrid offspring, is labeled as the probe, and the unlabeled DNA from other species as a blocking agent [25]. In this system, the labeled probe, especially the specific dispersed repetitive sequences, can hybridize to only one of the two sets of chromosomes to identify its origin [26]. The introduction helps to build a bridge between the cytological and molecular approaches and provide a useful tool for analyzing the genome structure of polyploid species and hybrid plants, especially distinguishing between parental genomes in interspecific plant hybrids without genomic background requirement [27,28]. Similar to GISH, fluorescence in situ hybridization (FISH) is also an effective method developed for chromosome analysis, which further helps to locate the same chromosomes or specific loci in a different set of polyploids, thus determining the copy numbers of a specific locus in polyploid plants [29]. FISH analysis may provide more details on nucleolar organizing region and ploidy when 45S and 5S ribosomal DNAs (rDNAs) are used as probes, respectively, although disputes still occurred on the relationship of 5S rDNA and ploidy [30,31,32]. rDNA is a highly conserved tandem repeat sequence that ubiquitously exists in plants as well as in bacteria and animals, while 45S and 5S belong to the major and minor gene classes, respectively, encoding different molecular sizes of rRNAs [33,34]. Therefore, the combination of GISH and FISH may accurately identify the parental origin of specific loci or chromosomes in the hybrid offspring, which has been successfully applied in ornamental plants as an efficient molecular cytogenetic tool for improving interspecific hybridization-based breeding, especially for the identification and transfer of specific genes from alien to native species [24].

All the nine wild tree peony species in *Paeonia* L. and their cultivars are diploid (2n = 2x = 10), while herbaceous peony has a complex genetic background with both diploids (*P. lactiflora* and *P. obovata*) and tetraploids (*P. officinalis* and *P. mairei*) (2n = 4x = 20) occurring in different species and some triploids (2n = 3x = 15) in the hybrid cultivars from the parents with different ploidies [35]. All the reported Itoh hybrid varieties are triploid, and for most, the maternal parent is the diploid *P. lactiflora*, while for all, the paternal parent is tree peony, with most as diploid *P. delavayi* var. *lutea* or its hybrid. In this study, 30 peony species or cultivars, including six in sect. *Moutan*, six in sect. *Paeonia*, and 18 Itoh hybrids were selected for karyotype analysis. Four representative Itoh hybrids were selected for GISH and FISH determination to further assess the genetic relationship between Itoh hybrids and their long-distant parents.

## 2. Results

### 2.1. Genome Size and Ploidy Analysis of Different Materials in Paeonia L.

**Figure 1:**
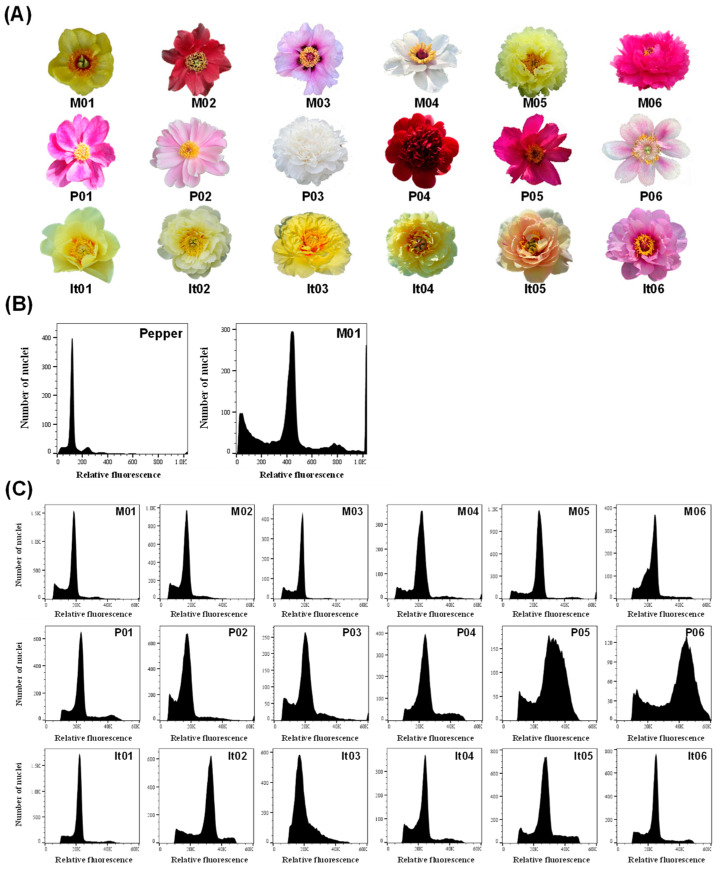
Flow cytometry analysis of the three taxa in *Paeonia* L., including sect. *Moutan* (M01–06), sect. *Paeonia* (P01–06), and Itoh hybrid (It01–06). (**A**) Morphology of the peonies; (**B**,**C**) flow cytometric histograms of pepper cv. *Zunla-1* and various of peonies. The names of the peony species and varieties are shown in Table 1.

In this study, the relative ploidy and genome size of 18 representative peony materials of three taxa, including sect. *Moutan* (M01–M06), sect. *Paeonia* (P01–P06), and Itoh hybrids (It01–It06) was first estimated (Table S1). The flow cytometric measurements showed that all the materials had clear and sharp peaks, except for P05 and P06 with much wider peaks (Figure 1). To compare the ploidy, the reading number of M01 in flow cytometry was set as 2.00, and the relative values in sect. *Moutan* ranged from 1.78 to 2.54, with both cultivated varieties (M05 and M06) having the equal and highest value. The relative values in sect. *Paeonia* were between 1.73 and 3.47, where P05 and P06 had the highest values. The relative values in Itoh hybrids ranged from 2.51 to 3.65. Concerning the genome size, it ranged from 10.77 Gb (P03) to 22.72 Gb (It01) with the pepper cv. *Zunla-1* as a standard (Table 1).

**Figure 2:**
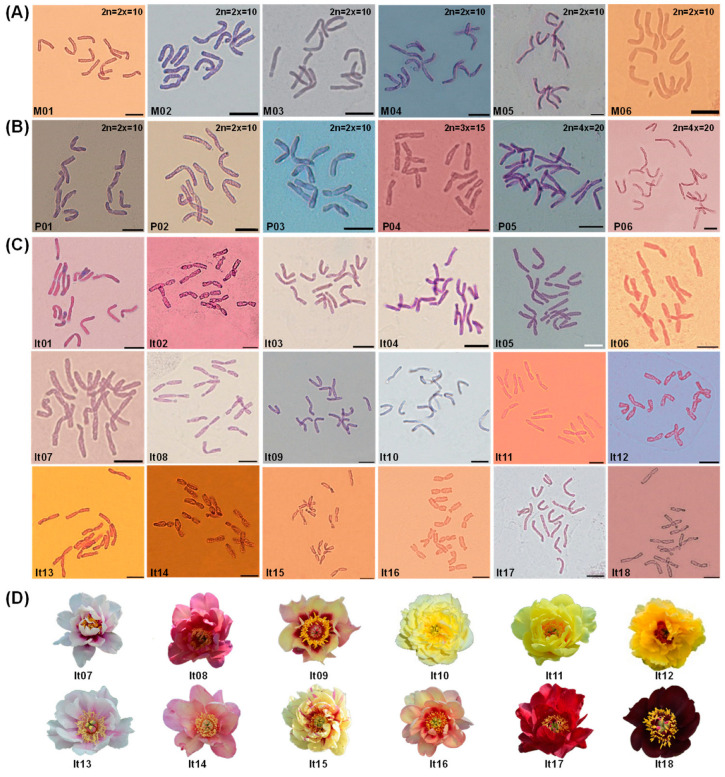
Metaphase chromosomes of 30 peony materials in (**A**) sect. *Moutan*, (**B**) sect. *Paeonia*, (**C**) Itoh hybrids, and (**D**) morphology of Itoh hybrids It07–It18. The ploidy of materials in (**A**,**B**) are shown on the upper right of each image. The codes in (**A**–**D**) represent the material names as shown in Table 1 and Table 2; bar = 10 μm.

The metaphase chromosomes of all the 30 peony materials were then determined. The basic chromosome number of the tested peonies was *x* = 5. All the materials in sect. *Moutan* were diploid (2n = 2x = 10, M01–06), while the plants in sect. *Paeonia* showed various ploidies, including diploid (2n = 2x = 10, P01–03), triploid (2n = 3x = 15, P04), and tetraploid (2n = 4x = 20, P05–06) (Figure 2A,B). The 18 Itoh hybrids, including the six varieties shown in Table 1, were triploids with 15 chromosomes (2n = 3x = 15, It01–18) (Figure 2C,D).

### 2.2. Karyotype Analysis of Peonies among Different Taxa

**Figure 3:**
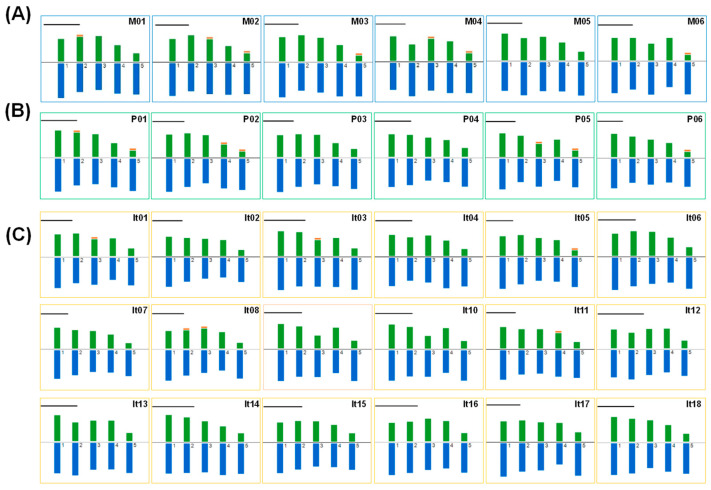
Chromosome idiograms of 30 species or varieties in *Paeonia* L. of (**A**) sect. *Moutan*, (**B**) sect. *Paeonia*, and (**C**) Itoh hybrids. The orange fragment on the top of partial chromosomes indicates satellites. The names of each code on the upper right of each image are shown in Table 1 and Table 2; bar = 10 μm.

The results showed that all the tested peonies had karyotype 2A. The karyotype formula in sect. *Moutan* was 6m + 2sm + 2st with 1–4 satellites, except for M05, the ‘High Noon’, whose formula was 8m + 2st and had no satellite. There were three karyotype formulas in sect. *Paeonia*, including 6m + 2sm + 2st (P01–03), 10m + 3sm + 2st (P04), and 12m + 4sm + 4st (P05–06), where only P03 and P04 had no satellite. For Itoh hybrids, 16 varieties had the same karyotype formula of 9m + 3sm + 3st, with only four varieties (It01, It03, It08, and It11) having 1–2 satellites, while It06 and It15 had the formulas of 12m + 3st and 10m + 3sm + 2st, respectively, both of which had no satellite (Figure 3; Table 2).

**Figure 4:**
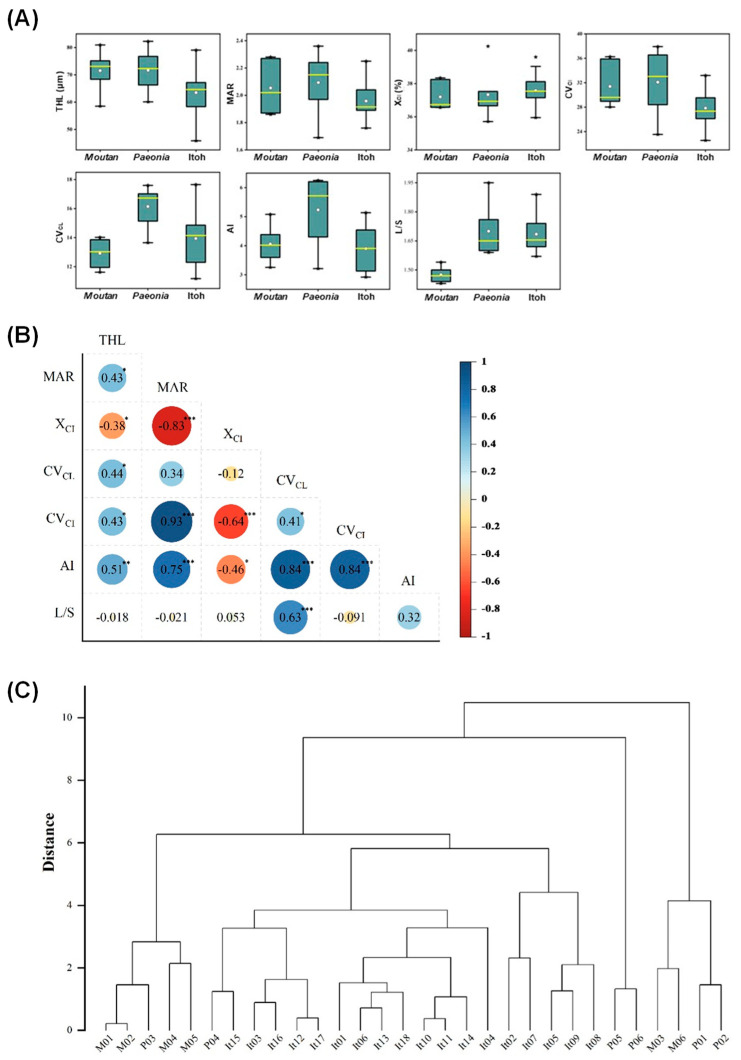
Comparison of karyological diversity among different peony species or varieties in sect. *Moutan*, sect. *Paeonia*, and Itoh hybrids. (**A**) The range and distribution of seven chromosomal parameters. Horizontal lines and hollow circles in the box represent median and mean values, respectively [50% of data are within the box, and 99% are within the bar (± SD)]. The asterisks represent data outside the box. (**B**) Pearson correlation analysis of seven chromosomal parameters shown in (**A**); the asterisks *, **, and *** indicate a significant correlation at the 0.05, 0.01, and 0.001 levels, respectively. (**C**) Hierarchical clustering analysis of the 30 peonies based on the seven chromosomal parameters shown in (**A**). The names of 30 peonies and categories of seven parameters are shown in Table 1 and Table 2.

The basic morphometric parameters of the chromosomes in these materials were also measured. The total haploid length of chromosomes (THL) varied from 45.84 μm (It12) to 82.25 μm (P06). The mean arm ratio (MAR) ranged from 1.69 (P04) to 2.28 (M03), while the mean centromeric index (X_CI_) was between 35.71% (P02) and 40.25% (P04) based on the centromere position. The minimum and maximum values of the coefficient of variation of chromosome length (CV_CL_) were 11.18 (It03) and 17.65 (It02), respectively. The minimum and maximum values of centromeric index (CV_CI_) were 22.56 (It15) and 37.91 (P02), respectively. The asymmetry index (AI) ranged from 2.92 (It15) to 6.25 (P02), and the ratio of the longest to short chromosomes (L/S) ranged from 1.43 (M05) to 1.95 (P06) (Table 2). The CV_CL_ and AI of Itoh hybrids and sect. *Paeonia* were close; the L/S of Itoh hybrids and sect. *Moutan* were also close; the other four parameters had no preference to any taxon (Figure 4A). The correlation among the seven parameters was further analyzed. Ten pairs were positively correlated with the highest value of MAR vs. CV_CI_, followed by AI vs. both CV_CL_ and CV_CI_. However, four pairs were negatively correlated with the order of MAR vs. X_CI_, X_CI_ vs. CV_CI,_ etc. (Figure 4B). A karyotype evolution map was then constructed based on the results in Table 2. The map showed that most materials in sect. *Moutan* and sect. *Paeonia* were clustered together, except for *P.* sp. ‘Red Charm’ (P04), the only triploid, which was closer to Itoh hybrids with the same ploidy. In addition, Itoh hybrids were not significantly correlated with sect. *Moutan* or sect. *Paeonia* (Figure 4C).

### 2.3. GISH Analysis of Peonies among Different Taxa

**Figure 5:**
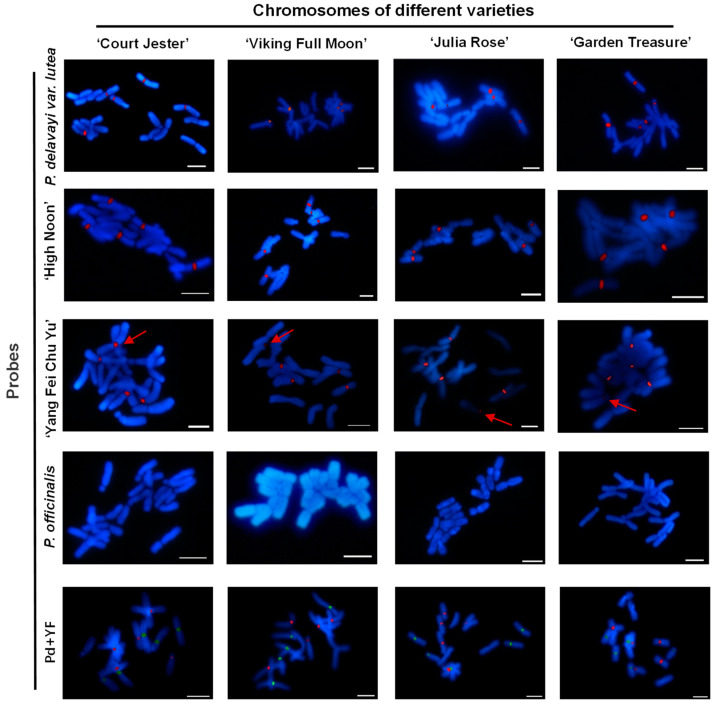
Comparative GISH mapping of four representative Itoh hybrids using gDNA of selected species or varieties as probes. Pd + YF indicates the mixed probes with *P. delavayi* var. *lutea* (green) and ‘Yang Fei Chu Yu’ (red). The red arrows indicate the labeled chromosomes with weak signals. Bar = 10 μm.

Four representative Itoh hybrid varieties [‘Court Jester’ (It09); ‘Viking Full Moon’ (It03); ‘Julia Rose’ (It08); and ‘Garden Treasure’ (It04)] were selected for GISH determination with the probes from *P. delavayi* var. *lutea* (M01; paternal parent of most Itoh hybrids); *P. × lemoinei* ‘High Noon’ (M05; hybrid progeny of M01; cultivated variety); *P. lactiflora* ‘Yang Fei Chu Yu’ (P03; variety of *P. lactiflora* as the maternal parent of most Itoh hybrids; and closer to Itoh hybrids in karyotype evolution map); and *P. officinalis* (P06; a tetraploid in sect. *Paeonia*) to further assess the parental origin of the chromosomes (Table S1). The M01; M05; and P03 probes labeled five chromosomes for every Itoh hybrid; whereas P06 labeled non-chromosomes on each Itoh hybrid. The mixed probes of M01 and P03 were used with different colors; and the results showed no overlapping of chromosomes between them in all the four Itoh hybrids (Figure 5). *P. suffruticosa* ‘Luo Yang Hong’ (M06) and *P. lactiflora* ‘Bai Shao’ (P02) were also used as probes for GISH determination, and no signal was detected on any of the four Itoh hybrids (data not shown).

**Figure 6:**
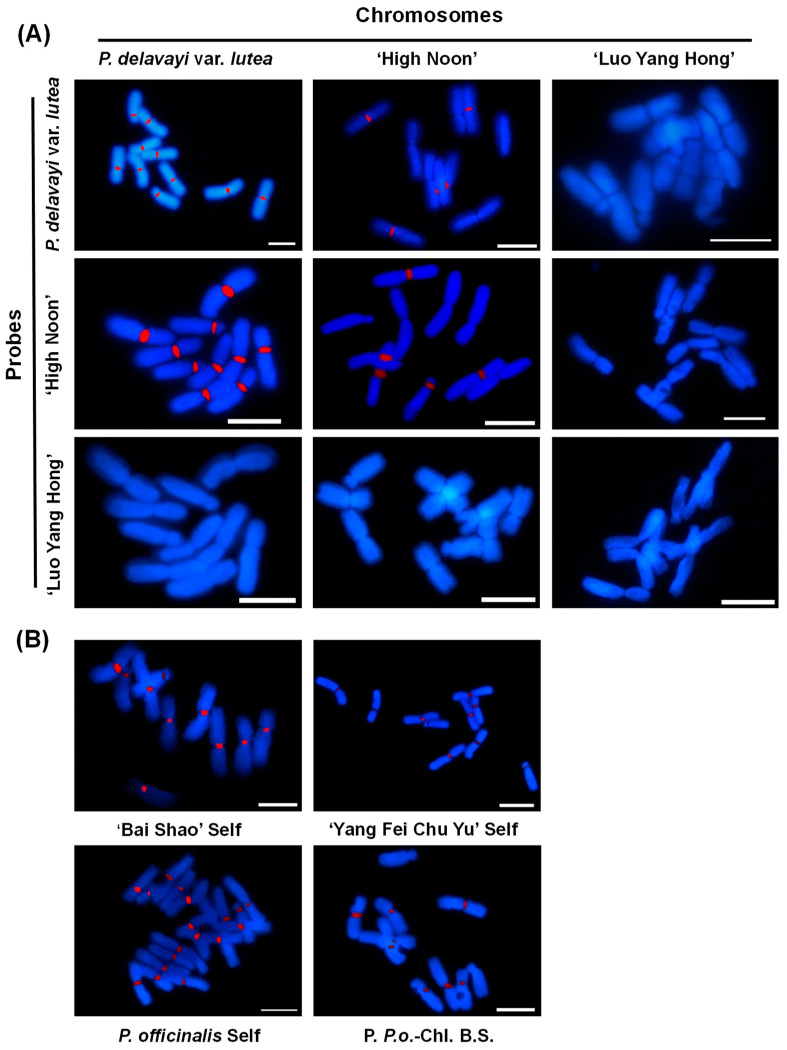
GISH analysis of mitotic chromosome pairings by pairwise hybridization in (**A**) sect. *Moutan* and (**B**) sect. *Paeonia*. Self refers to a situation where the material is used both as probe and chromosomal slide; P. *P.o*-Chl. B.S. means *P. officinalis* and ‘Bai Shao’ are used as probe and chromosomal slide, respectively. Bar = 10 μm.

Different peony taxa had different GISH signals. As a result, a pairwise hybridization was set among M01, M05, and M06 to test the current GISH system and plant materials (Table S1). Ten and five chromosomes were labeled on M01 and M05, respectively, while the M06 probe had no signal on any material (Figure 6A). The effect of self and mutual hybridizations in sect. *Paeonia* was also determined. All the chromosomes were labeled using their probes. Additionally, the P06 probe labeled at least eight chromosomes of P02 (Figure 6B).

**Figure 7:**
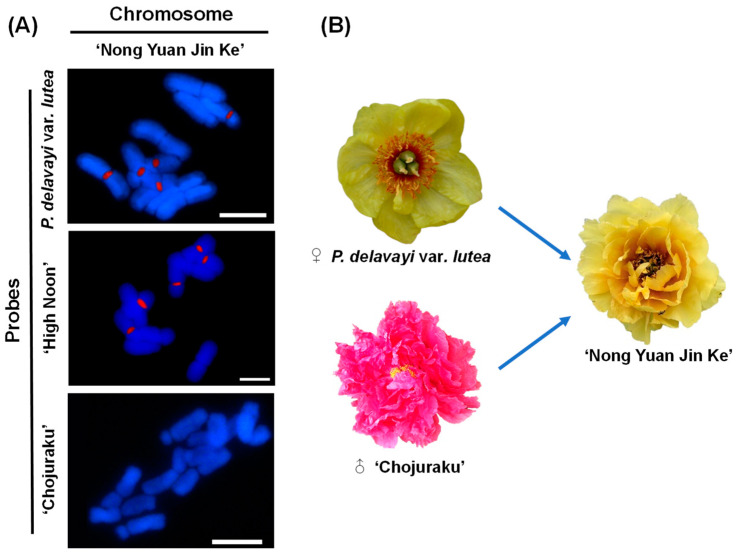
GISH analysis of mitotic pairings in ‘Nong Yuan Jin Ke’ using *P. delavayi* var. *lurea*, ‘High Noon’ and ‘Chojuraku’ as gDNA probes. (**A**) GISH results, bar = 10 μm; (**B**) morphylogy of ‘Nong Yuan Jin Ke’ and its parents.

It was difficult to obtain the parents’ information of the tested materials in Figure 6 (Table S1). Therefore, *P.* *suffruticosa* ‘Nong Yuan Jin Ke’ (NY), one of the newly cultivated varieties from the hybridization of M01 and *P.* *suffruticosa* ‘Chojuraku’, was used for GISH determination with the addition of M05, an offspring of M01. The M01 and M05 probes labeled five chromosomes on NY, while ‘Chojuraku’ had no signal on NY (Figure 7).

### 2.4. FISH Analysis of Peonies among Different Taxa

**Figure 8:**
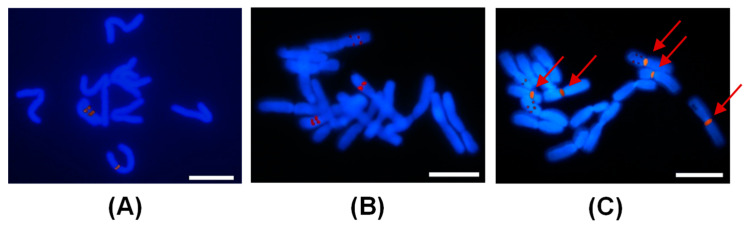
5S rDNA-FISH analysis of mitotic chromosome pairings in (**A**) ‘Luo Yang Hong’ and (**B**) ‘Garden Treasure’, and (**C**) the combination of 5S rDNA-FISH and GISH in the same cell of ‘Julia Rose’ with *P. delavayi* var. *lutea* as the GISH probe with five red arrows indicating the GISH signals.

The genus *Paeonia* has no high-quality genomic data for reference. Therefore, the 5S probe from maize (*Zea mays*) was used for FISH analysis [37]. Two and three chromosomes were labeled in diploid ‘Luo Yang Hong’, and triploid ‘Garden Treasure’ and ‘Julia Rose’, respectively, which were identical to their ploidies. GISH was also applied in the same cell of ‘Julia Rose’ using gDNA of *P. delavayi* var. *lutea* as the probe. Only one chromosome was co-labeled by both FISH and GISH (Figure 8).

## 3. Discussion

Flow cytometry is an effective cytogenetic method for estimating the genome size and ploidy in plants [38]. Flow cytometry has been used to assess nuclear DNA contents in tribe Leucocoryneae, and it showed a monoploid genome size variation among 23 species. The changes in genome size were associated with the diversification of lineages, supporting the hypothesis that the high rate of diversification is related to the ability to benefit from changes in genome size, such as polyploidy and genome rearrangements [39,40]. Flow cytometry of *Sorbus* also showed that the genome size of eight Chinese native species could infer their ploidy levels [41]. In this study, the relative genome sizes of M05 and M06, two cultivated varieties in sect. *Moutan*, were bigger than that of wild species, probably due to genome rearrangements. The changes in genome size may improve their ornamental characteristics. In contrast, the genome sizes of P02 and P03, two cultivated varieties in sect. *Paeonia*, were smaller than that of the wild species, probably due to the effect of chromosomal reorganizations [42]. The monoploid genome size of the six varieties in Itoh hybrids was similar to the other two taxa, indicating that they may improve their survival rate by increasing polyploidy since it is difficult to obtain offspring through distant hybridization between tree peony and herbaceous peony.

In addition to flow cytometry, karyomorphology is also widely used to compare the diversity and ploidy among different plants, especially the relationship and origin of different materials within the same genus [41]. In a study of three endemic plants on the Qinghai–Tibet Plateau, the analysis of chromosome number and karyotype verified the placement of the genera *Anzhengxia* and *Shangrilaia* in tribe Euclidieae DC. (Brassicaceae) [43], consistent with the previous presumption via nrITS sequencing [44]. Karyotype analysis among different species in the prayer-plant family (Marantaceae) showed a strong variation in chromosome number and size, possibly due to dysploid variation, polyploidy, and hybridization [45]. The karyomorphological features of seven species in all five genera of Nyssaceae showed that the chromosome number was similar within the same genus. Further detailed data analysis suggested that Nyssaceae are of hexaploid origin with the basic number x = 7 [46]. In wheat, the presence of addition chromosomes was also confirmed in alien disomic addition lines based on karyotype analysis [14]. In the current study, chromosome counting and comparison showed that all the Itoh hybrids were triploids. However, further karyotype analysis, correlation analysis, and hierarchical clustering could not identify the parental origin of each set of chromosomes. Both known parents of all the Itoh hybrids were diploids (Table S1). The genomic background of Itoh hybrids is complex due to the individual differences in genome size. Therefore, determining the origin of each set of chromosomes may help in constructing their genetic relationship maps. In addition, karyotype has been wildly applied in humans and animals for chromosome analysis [20,21], which can be good references in our future studies.

GISH, as a molecular cytogenetic technique, has been widely used for hybrid identification in plants. It uses the total genomic DNA as a probe, and it is suitable for materials without genomic backgrounds [47]. GISH also allows the observation of recombination or alterations between different genomes [48]. The relationships of different species in the Pooideae subfamily of Poaceae can be determined via GISH analysis of the whole chromosomes or partial regions. The unlabeled chromosome regions can also be identified, indicating that disparate pathways for chromosome differentiation are associated with species-specific sequences [49]. The hybrid paternity information of *Passiflora* is essential for determining germplasm origin via GISH. GISH can also allow the visualization of recombination between the homeologous chromosome and the introgression of sequences of interest [47].

In this study, the probes for GISH in sect. *Moutan* and sect. *Paeonia* labeled five chromosomes in Itoh hybrids without overlapping, while the other five chromosomes were not labeled (Figure 5; data not shown). The accuracy and efficiency of GISH on peony were determined using the probes and chromosomes from the same taxon and same material to eliminate potential technical effects. Interestingly, the cultivated variety of ‘Luo Yang Hong’ did not detect any signal either as the probe or the chromosome slide. However, ‘High Noon’ and *P. delavayi* var. *lutea* as probes labeled half of the chromosomes in ‘High Noon’. Both parents of ‘Luo Yang Hong’ were cultivated varieties, while ‘High Noon’ was from half wild and half cultivated tree peonies. Therefore, these results suggest that it may be difficult to label the chromosomes of tree peony from the cultivated varieties through the current GISH method, probably due to their huge and complex genomes compared with the wild species. The GISH results from the same material and taxon for the materials in sect. *Paeonia* met our expectations, indicating that only one set of the chromosomes in Itoh hybrids was from sect. *Paeonia*. The chromosomes of our cultivated tree peony were also used for GISH determination since it had clear parental information. The cultivated parent had no signal, further confirming that GISH cannot label chromosomes of the cultivated tree peony. These results indicate that of the three sets of chromosomes in Itoh hybrids, two may be from tree peony and one from herbaceous peony. One of two sets from the tree peony may be from the wild species, and the other from the cultivated variety. In addition, the chromosomes of *Paeonia* plants are large, and possibly contain many repetitive sequences; however, in our results, the GISH signals were mainly concentrated in the centromere region, which was similar to the reports of *Brassica* and *Setaria* species, probably due to the high concentration of repeats in the centromere region [50,51,52].

FISH is also extensively used as one of the molecular cytogenetic techniques for paternity confirmation in hybrids, especially for determining specific chromosomes using specific probes, such as 45S and 5S rDNA [33,34]. FISH provided unique chromosome markers from each parent in *Passiflora* using rDNA probes, thus facilitating the recognition of each genome genitor in the hybrids [47]. FISH revealed one 5S locus and eight 45S loci in *Lablab purpureus* using rDNA probes, thus helping to identify the prometaphase chromosome pair combined with the CPD and DAPI^+^ bands, as well as chromosome measurements [53]. Most 5S rDNA signals are located in subterminal chromosome regions in *Chrysanthemum* based on the determination of Oligo-FISH, and the number of 5S rDNA sites is significantly associated with ploidy [54].

There are few FISH-related cytogenetic studies on *Paeonia*. Most studies focused on chromosomal structural rearrangement and rDNA loci evolution using 45S rDNA as probes, which mostly labeled more than one rDNA site [55,56]. However, no study has reported on 5S rDNA-based FISH in *Paeonia*. In this study, we found that the number of 5S rDNA loci was consistent with the ploidy in all the three tested materials, thus we presumed it may be used for ploidy assessment in *Paeonia*, although more directed evidence is still needed. In addition, only one chromosome in the Itoh hybrid was co-labeled by GISH and FISH, indicating that the five chromosomes labeled by GISH were in the same set.

## 4. Materials and Methods

### 4.1. Plant Material

In this study, all the *Paeonia* materials, including those in sect. *Moutan*, sect. *Paeonia*, and Itoh hybrids, were obtained from the Yanqing Cultivation Base of Beijing, China (116°15′51″ N, 40°33′32″ E; at 673 m a.s.l.). The detailed information is listed in Table S1.

### 4.2. Flow Cytometric Measurement

The flow cytometric measurement was conducted as described by [57], and the genome sizes of various peonies were estimated with pepper (*Capsicum annuum* L. cv. *Zunla-1*) as a standard [36], according to [58,59]. Briefly, the young leaves from different individual plants were separately collected and washed twice using distilled water. About 0.5 cm^2^ of healthy leaf tissue was excised and placed into a plastic Petri dish on ice, then cut into small pieces using a sharp razor blade and put in 250 μL of ice-cold extraction buffer (CYStain UV Precise P Nuclei Extraction Buffer) for 30 s. Staining buffer (1000 μL) (CYStain UV Precise P Staining Buffer) was then added to the sample and mixed. The homogenate was filtered using a 50 μm nylon net and transferred into a sample tube. The samples were incubated on ice in the dark for 5–10 min. CyFlow Counter flow cytometry (Sysmex Partec, Goerlitz, Germany) was then used for ploidy detection. FlowJo (BD Biosciences, San Jose, CA, USA) was used to analyze the data.

### 4.3. Karyotype Analysis

Actively growing root tips (1–3 cm) from mature plants were cut from 9:00 am to 11:00 am in early March and pretreated with 0.1% cycloheximide in the dark for 8–10 h. The samples were fixed in Carnoy’s solution (ethanol:acetic acid = 3:1) for 12 h, then stored in 75% ethanol at 4 °C until for further use. Slices were prepared as follows: the root tips were hydrolyzed in 1 M HCl at 60 °C for 10 min, washed thrice with distilled water, placed on glass slides, and cut into pieces using a sharp knife. The samples were then stained with carbol fuchsin (Solarbio, Beijing, China) for 15–20 min. Olympus BX53F (Olympus, Tokyo, Japan) was used for obtaining images (at least five metaphase cells per sample).

Karyotype-related parameters were calculated as described by [60,61], and presented as follows: total haploid (monoploid) length of chromosome set (THL) = sum of total chromosome length; relative length (RL) = related chromosome length/total chromosome length; mean arm ratio (MAR) = mean (long arms/short arms); mean centromeric index [X_CI_ (%)] = mean [short arms/(long arms + short arms) × 100%]; coefficient of variation of chromosome length (CV_CL_) = standard deviation of chromosome length/mean chromosome length; coefficient of variation of centromeric index (CV_CI_) = standard deviation of CI/mean CI, where CI means the ration of short arm to the whole chromosome, which determines the relative position of centromeres; asymmetry index (AI) = CV_CL_ × CV_CI_; ratio of the longest/short chromosomes (L/S) = the longest chromosome length/the shortest chromosome length. For data analysis, about 3–5 individuals and 3–5 metaphase spreads per individual were considered.

### 4.4. GISH and FISH

GISH and FISH were performed as described by [62] with minor modifications. Briefly, the roots underwent a pre-hypotonic treatment with 0.075 mol L^−1^ KCl for 30 min, followed by digestion using a mixture of 2% Cellulase Onozuka RS (Yakult, Tokyo, Japan) and 0.5% Pectolyase Y-23 (Yakult, Tokyo, Japan) at 37 °C for 100 min. The meristems were washed with distilled water, followed by hypotonic treatment for 30 min. The meristems were then squashed in Carnoy’s solution on a glass slide using fine-pointed forceps and flame-dried. The slides were stored at –20 °C before further treatment.

The probe preparation of GISH was conducted as follows: the genomic DNA from young leaves of the selected plants was isolated via the CTAB method [63]. The samples were labeled with digoxigenin-11-dUTP or biotin-16-dUTP (Roche Diagnostics, Risch-Rotkreuz, Switzerland) via nick translation at 15 °C for 120 min (final DNA concentration, 200 ng μL^−1^; fragment size, 200–500 bp), as described by [64]. The probe used for FISH was obtained from Prof. Weiwei Jin at the China Agriculture University with the final concentration of 200 ng μL^−1^.

For hybridization, the slides with cytological preparations were dried at 65 °C for at least 1 h. The slides were then denatured with 100 μL (70% formamide, 10% 20× SSC, 20% ddH_2_O) at 82 °C for 10 min, dehydrated in precooled ethanol (70, 95, and 100%), and air-dried. Hybridization mixture containing 50% (*v*/*v*) formamide, 10% (*w*/*v*) dextran sulfate, 2× SSC, 2~4 μL labeled probe, and 2~4 μL salmon sperm DNA (Solarbio, Beijing, China) was applied to a selected area of each slide. The solution was treated at 95 °C for 10 min and instantly quenched in ice before hybridization. The hybridization solution was then applied to the slide, covered with a cover glass, and incubated at 37 °C for 12 h. The samples were washed twice after hybridization by 2× SSC solution at room temperature for 5 min each, and then incubated in a Coplin jar at 42 °C containing 2× SSC and washed for 10 min. Then, the slides were washed by 2× SSC solution at room temperature for 5 min and finally washed by 1× PBS solution at room temperature for 5 min.

Anti-digoxingenin-rhodamine (100 μL) (red color) (Roche, Basel, Switzerland) or anti-boitin-FITC (blue color) (Avidin-FITC from egg white, Sigma, St. Louis, MO, USA) was added to the slides [1:100 in TNB buffer (0.1M Tris-HCl, pH 7.5, 0.15 M NaCl, and 0.5% blocking reagent)] at 37 °C for 1 h for detection. The samples were washed thrice using 1× PBS (for 5 min each) at 37 °C to remove excess antibody. The slides were subsequently mounted and counterstained with DAPI/Vectashield (Vector Laboratories. Burlingame, CA, USA). Olympus BX53F (Olympus Tokyo Japan) was used to obtain images. The Adobe Photoshop CS6 was used for brightness and contrast adjustments.

### 4.5. Data Analysis

MATO was used to draw a mean haploid ideogram based on the chromosome length [65]. Hierarchical clustering was performed to determine the karyological relationships among different materials. OriginPro 2019b (OriginLab Software, USA) was used to draw the correlation plot and boxplot.

## 5. Conclusions

In this study, the monoploid genome sizes of selected Itoh hybrids showed individual differences. However, genome sizes were not significantly different between sect. *Moutan* as the paternal parent and sect. *Paeonia* as the maternal parent. Further karyotype analysis could not identify the parental origin of chromosomes of Itoh hybrids. GISH and FISH verifications suggested that of three sets of chromosomes in Itoh hybrids, two were from the paternal parent, and one was from the maternal parent. One of the two sets from the paternal parent could be from wild species and the other from the cultivated variety. Meanwhile, the 5S rDNA-based FISH was first applied in *Paeonia* for ploidy assessment. This work may give more insights into improving Itoh hybrid resources.

## Figures and Tables

**Table 1:** Information and relative genome size of 18 species or varieties in *Paeonia* L.

Taxa	Code	Name	Indication ^a^	Relative Ploidy ^b^	Genome Size (Gb) ^c^
Sect. *Moutan*	M01	*P. delavayi* var. *lutea*	18,823	2.00	12.45
	M02	*P. delavayi*	16,778	1.78	11.08
	M03	*P. rockii*	17,966	1.91	11.89
	M04	*P. ostii*	21,475	2.28	14.19
	M05	*P. × lemoinei* ‘High Noon’	23,902	2.54	15.81
	M06	*P. suffruticosa* ‘Luo Yang Hong’	23,928	2.54	15.81
Sect. *Paeonia*	P01	*P. veitchii*	22,341	2.37	14.75
	P02	*P. lactiflora* ‘Bai Shao’	16,945	1.80	11.21
	P03	*P. lactiflora* “Yang Fei Chu Yu’	16,271	1.73	10.77
	P04	*P.* sp. ‘Red Charm’	19,997	2.12	13.20
	P05	*P. mairei*	31,416	3.34	20.79
	P06	*P. officinalis*	32,695	3.47	21.60
Itoh hybrid	It01	‘Going Bananas’	34,352	3.65	22.72
	It02	‘Bartzella’	32,167	3.42	21.29
	It03	‘Viking Full Moon’	24,582	2.61	16.25
	It04	‘Garden Treasure’	23,634	2.51	15.62
	It05	‘Prairie Charm’	26,888	2.86	17.80
	It06	‘Morning Lilac’	24,645	2.62	16.31

^a^ Reading number on flow cytometry; ^b^ the size of *P. delavayi* var. *lutea* was set as 2.00; ^c^ based on the genome size of pepper (3.35 Gb) as a standard [36].

**Table 2:** Collecting information of materials and cytogenetics data.

Taxon	Code	Name	Ss’C	Ploidy	HKF	THL (μm)	RL (%)	MAR	X_CI_ (%)	CV_CL_	CV_CI_	AI	L/S
Sect. *Moutan*	M01	*P. delavayi* var. *lutea*	2A	2n = 2x = 10	6m * + 2sm + 2st	68.38	15.87~23.11	1.87	38.25	13.86	28.95	4.01	1.50
	M02	*P. delavayi*	2A	2n = 2x = 10	6m ** + 2sm + 2st **	73.90	15.66~22.26	2.02	36.57	13.85	29.09	4.03	1.46
	M03	*P. rockii*	2A	2n = 2x = 10	6m + 2sm + 2st *	75.13	15.45~22.90	**2.28**	36.56	14.02	36.26	5.08	1.54
	M04	*P. ostii*	2A	2n = 2x = 10	6m * + 2sm + 2st *	80.94	15.79~21.77	2.02	36.70	11.95	30.10	3.60	1.44
	M05	*P. × lemoinei* ‘High Noon’	2A	2n = 2x = 10	8m + 2st	72.31	16.66~22.02	1.86	38.34	11.62	28.02	3.26	**1.43**
	M06	*P. suffruticosa* ‘Luo Yang Hong’	2A	2n = 2x = 10	6m + 2sm + 2st *	58.51	16.00~22.56	2.27	36.76	12.21	35.89	4.38	1.48
Sect. *Paeonia*	P01	*P. veitchii*	2A	2n = 2x = 10	6m * + 2sm + 2st *	66.28	16.06~24.81	2.20	37.53	16.97	36.55	6.20	1.60
	P02	*P. lactiflora* ‘Bai Shao’	2A	2n = 2x = 10	6m + 2sm ** + 2st *	72.74	15.64~23.95	2.36	**35.71**	16.48	37.91	**6.25**	1.63
	P03	*P. lactiflora* “Yang Fei Chu Yu’	2A	2n = 2x = 10	6m + 2sm + 2st	76.75	16.30~23.76	1.97	36.97	15.14	28.41	4.30	1.59
	P04	*P.* sp. ‘Red Charm’	2A	2n = 3x = 15	10m + 3sm + 2st	60.11	16.94~23.42	**1.69**	**40.25**	13.65	23.54	3.21	1.67
	P05	*P. mairei*	2A	2n = 4x = 20	12m + 4sm * + 4st *	71.81	16.31~25.37	2.10	36.89	17.02	32.48	5.53	1.76
	P06	*P. officinalis*	2A	2n = 4x = 20	12m + 4sm + 4st ***	**82.25**	15.61~24.96	2.24	36.66	17.60	33.58	5.91	**1.95**
Itoh hybrid	It01	‘Going Bananas’	2A	2n = 3x = 15	9m + 3sm ** + 3st	69.70	16.17~23.73	1.85	38.48	14.98	25.57	3.83	1.77
	It02	‘Bartzella’	2A	2n = 3x = 15	9m + 3sm + 3st	64.56	15.50~24.94	1.91	38.33	**17.65**	27.74	4.90	1.89
	It03	‘Viking Full Moon’	2A	2n = 3x = 15	9m * + 3sm + 3st	**58.31**	16.41~22.36	2.04	36.45	**11.18**	26.94	3.01	1.57
	It04	‘Garden Treasure’	2A	2n = 3x = 15	9m + 3sm + 3st	58.99	16.34~22.79	1.99	37.15	12.13	29.54	3.58	1.57
	It05	‘Prairie Charm’	2A	2n = 3x = 15	9m + 3sm + 3st *	79.05	16.32~23.68	2.25	35.95	14.84	32.19	4.78	1.62
	It06	‘Morning Lilac’	2A	2n = 3x = 15	12m + 3st	64.07	16.44~22.60	1.79	39.60	13.50	26.34	3.56	1.65
	It07	‘Cora Louise’	2A	2n = 3x = 15	9m + 3sm + 3st	73.35	15.72~24.74	2.09	36.83	17.14	29.97	5.14	1.74
	It08	‘Julia Rose’	2A	2n = 3x = 15	9m * + 3sm * + 3st	64.68	16.56~23.38	2.16	37.16	13.68	33.19	4.54	1.62
	It09	‘Court Jester’	2A	2n = 3x = 15	9m + 3sm + 3st	59.63	17.10~24.45	1.98	37.82	15.13	31.00	4.69	1.73
	It10	‘Lemon Dream’	2A	2n = 3x = 15	9m + 3sm + 3st	66.70	15.50~23.27	2.06	36.17	14.26	29.08	4.15	1.87
	It11	‘Sequestered Sunshine’	2A	2n = 3x = 15	9m + 3sm * + 3st	72.00	15.97~24.01	1.98	37.48	14.29	28.81	4.12	1.63
	It12	‘Yellow Crown’	2A	2n = 3x = 15	9m + 3sm + 3st	45.84	15.80~21.96	1.91	37.39	12.30	25.44	3.13	1.66
	It13	‘Ballarena de Saval’	2A	2n = 3x = 15	9m + 3sm + 3st	65.58	15.85~23.47	1.89	37.90	14.04	26.75	3.76	1.63
	It14	‘Sugar Plum Fairy’	2A	2n = 3x = 15	9m + 3sm + 3st	58.35	15.75~23.21	1.90	37.61	14.28	27.90	3.98	1.69
	It15	‘Lollipop’	2A	2n = 3x = 15	10m + 3sm + 2st	55.73	16.19~22.76	1.76	39.04	12.95	**22.56**	**2.92**	1.59
	It16	‘Magical Mystery Tour’	2A	2n = 3x = 15	9m + 3sm + 3st	54.54	16.15~21.69	1.92	37.27	11.49	26.12	3.00	1.67
	It17	‘Scarlet Heaven’	2A	2n = 3x = 15	9m + 3sm + 3st	67.17	17.19~23.26	1.89	37.66	12.29	25.05	3.08	1.65
	It18	‘Dark Eyes’	2A	2n = 3x = 15	9m + 3sm + 3st	64.69	16.03~23.60	1.87	38.12	14.86	26.90	4.00	1.77

Note: Ss’C: Stebbins’s classification; HKF: haploid karyotype formula, *, ** and *** mean one, two and three satellites, respectively; THL: total haploid (monoploid) length of chromosome set; RL: relative length; MAR: mean arm ratio; X_CI_: mean centromeric index; CV_CL_: coefficient of variation of chromosome length; CV_CI_: coefficient of variation of centromeric index; AI: asymmetry index; L/S: ratio of the longest/short chromosomes. The bold fond of data presents the maximum or minimum value in each catalog.

## Data Availability

The data used to support the findings of this study are available from the corresponding authors upon request.

## Supplementary Materials

The following supporting information can be downloaded at: https://www.mdpi.com/article/10.3390/ijms231911406/s1.
