# Supplementary material for: Karyotype Analysis, Genomic and Fluorescence In Situ Hybridization (GISH and FISH) Reveal the Ploidy and Parental Origin of Chromosomes in Paeonia Itoh Hybrids
Source: Int J Mol Sci. 2022 Sep 27;23(19):11406. doi: 10.3390/ijms231911406 (PMC9570356; doi:10.3390/ijms231911406)
Supplement: Supplementary file 1 [file ijms-23-11406-s001.zip › ijms-1932603-supplementary.pdf]

Table S1. Basic information of the materials involved in this study

Code	Name	Origin or parents' information*	Experiments conducted (filled by ●)			
			Flow cytometric analysis	Karyotype analysis	GISH	FISH
M01	*P. delavayi* var. *lutea*	Wild species collected from Yunnan, China	●	●	●	
M02	*P. delavayi*	Wild species collected from Yunnan, China	●	●		
M03	*P. rockii*	Wild species collected from Gansu, China	●	●		
M04	*P. ostii*	Wild species collected from Anhui, China	●	●		
M05	*P.* × *lemoinei* 'High Noon'	*P. delavayi* var. *lutea* ×*P. suffruticosa* cv.	●	●	●	
M06	*P. suffruticosa* 'Luo Yang Hong'	Traditional cultivar collected from Henan,	●	●	●	●
P01	*P. veitchii*	Wild species collected from Sichuan, China	●	●		
P02	*P. lactiflora* 'Bai Shao'	Common cultivar collected from Anhui, China	●	●	●	
P03	*P. lactiflora* "Yang Fei Chu Yu'	Traditional cultivar collected from Shandong,	●	●	●	
P04	*P.* sp. 'Red Charm'	*P. officinalis* 'Rubra Plena' × *P. lactiflora* cv.	●	●		
P05	*P. mairei*	Wild species collected from Xinjiang, China	●	●		
P06	*P. officinalis*	Wild species collected from the Netherlands	●	●	●	
It01	Going Bananas	Unknown ×Unknown		●		
It02	Bartzella	White *lactiflora* double × Reath hybrid (tree peony)		●		
It03	Viking Full Moon	*P. lactiflora* × Yellow *lutea* hybrid		●	●	
It04	Garden Treasure	*P. lactiflra*, cultivar × *P. Lutea* Hybrid Alice		●	●	●
It05	Prairie Charm	Miss America × *P. Lutea* Hybrid Alice Harding		●		
It06	Morning Lilac	Martha W. seeding × Golden Era. (*Lutea* hybrid)		●		
It07	Cora Louise	*P. lactiflora*, white double × Reath hybrid (tree peony)		●		
It08	Julia Rose	Unknown × Unknown		●	●	●
It09	Court Jester	*P. lactiflora* 'Martha W.' × D-256 Tria seedling		●	●	
It10	Lemon Dream	*P. lactiflora* 'Martha W.' × Golden Era. (*Lutea* hybrid)		●		
It11	Sequestered Sunshine	Miss American × D-74		●		
It12	Yellow Crown	*P. lactiflora* 'Kakoden' × *P. Lutea* Hybrid Alice		●		
It13	Ballerena de Saval	Unknown × Unknown		●		
It14	Sugar Plum Fairy	Unknown × Unknown		●		

It15	Lollipop	Anderson`s seedling × D-79		●	
It16	Magical Mystery Tour	Martha W. seeding × Golden Era. (*Lutea* hybrid)		●	
It17	Scarlet Heaven	Martha W. seeding × Thunderbolt		●	
It18	Dark Eyes	*P. lactiflora* ‘Mikado’ × *P. delavayi*		●	
/	*P. suffrulicosa* ‘Chojuraku’	Japanese cultivar collected from Henan, China			●
/	*P. suffrulicosa* ‘Nong Yuan Jin	*P. delavayi* var. *lutea* × *P. suffrulicosa* ‘Chojuraku’			●
/	Pepper cv. *Zunla-1*	Fresh leave obtained from Dr. Hailong Yu in CAAS	●		

*The parents’ information of Itoh hybrids is obtained from the online data of American Peony Society (https://www.americanpeonysociety.org) as maternal parent × paternal parent.
